# Employment of *L. paracasei* K5 as a Novel Potentially Probiotic Freeze-Dried Starter for Feta-Type Cheese Production

**DOI:** 10.3390/microorganisms7010003

**Published:** 2018-12-26

**Authors:** Antonia Terpou, Ioanna Mantzourani, Alex Galanis, Maria Kanellaki, Eugenia Bezirtzoglou, Argyro Bekatorou, Athanasios A. Koutinas, Stavros Plessas

**Affiliations:** 1 Food Biotechnology Group, Section of Analytical Environmental and Applied Chemistry, Department of Chemistry, University of Patras, GR-26500, Patras, Greece; m.kanellaki@upatras.gr (M.K.); abekatorou@upatras.gr (A.B.); a.a.koutinas@upatras.gr (A.A.K.); 2 Laboratory of Microbiology, Biotechnology & Hygiene, Faculty of Agricultural Development, Democritus University of Thrace, 68200 Orestiada, Greece; imantzou@agro.duth.gr (I.M.); empezirt@agro.duth.gr (E.B.); splessas@agro.duth.gr (S.P.); 3 Department of Molecular Biology and Genetics, Democritus University of Thrace, Alexandroupolis 68100, Greece; agalanis@mbg.duth.gr

**Keywords:** Feta-type cheese, *Lactobacillus paracasei* K5, starter culture, freeze-drying, probiotics, aromatic profile

## Abstract

In the present study, a novel potentially probiotic *Lactobacillus paracasei* strain, previously isolated from dairy products, was evaluated as a starter culture of Feta-type cheese production. Targeting industrial applications, the starter culture was applied as a ready-to-use freeze-dried culture that was either free or immobilized. The immobilized biocatalyst composed of *Lactobacillus paracasei* K5 cells absorbed within delignified wheat bran prebiotic carrier. All produced cheeses were compared with cheese manufactured by renin enzyme. Several parameters that affect acceptability, quality and shelf-life of Feta-type cheese were investigated, including microbial populations, physicochemical characteristics and cheese volatiles through 90 days of ripening and storage. Survival of *L. paracasei* K5 remained in high levels (≥6.0 log cfu/g) after the 90th day of cheese production, as recorded by combining microbiological enumeration and strain-specific multiplex PCR analysis. The use of the freeze-dried novel starter culture (free or immobilized) enhanced the aromatic profile of Feta-type cheeses. Finally, the use of the potentially synbiotic immobilized biocatalyst further improved aromatic characteristics of produced cheese and decrease of possible spoilage or pathogenic microorganisms. These findings indicate the potential industrial use of freeze-dried *L. paracasei* K5 as starter culture for the production of good-quality functional Feta-type cheese.

## 1. Introduction

Nowadays, functional food products have drowned the interest of scientific community, food and nutraceutical industry as they claim to promote human health by improving consumers’ physical and mental well-being [1,2]. Specifically, functional foods are claimed as such since they contain health-promoting components which go beyond the traditional nutrients [1]. One of the most successful ways in which foods can be modified to become functional is by the addition of probiotic bacteria. A probiotic food is a processed food product which contains viable probiotic bacteria contained in a sufficient concentration within the food matrix [3]. The food industry, targeting to meet the market’s needs, has successfully provided probiotic food to the consumers upsurge demand over the last few years while it has been forecasted that the global sales market of functional food will exceed $300 billion by 2020 (Source: Research and Markets) [4].

As a result of consumers demand on better quality and added value functional food products, an upsurge interest on exploiting and applying novel functional cultures has been developed during the last decade in the food industry. The main scope of this trend is the production of novel food products with upgraded sensorial characteristics, probiotic properties, enhanced shelf-life, and higher nutritional value [1]. To this aim, various starter cultures have been used, mainly belonging to Lactic Acid Bacteria (LAB) and Bifidobacterium genus, as they play a key role in the production of functional dairy products. Specifically, probiotics would be excellent starter cultures contributing not only to possible health benefits, but also to flavour, aroma, texture and to an upgraded nutritional value by thereby helping determine unique products’ characteristics [5]. Although the selection criteria for probiotic strains for human consumption should privilege/favour microorganisms of human origin, currently many non-starter LAB (NSLAB) have been isolated from a variety of ripened cheeses and used in commercial probiotic products [6]. Lactobacilli species play a key role in the dairy industry regarding fermented products while they can be detected within a major part of gut microbiota [7]. In addition, lactobacilli that are isolated from fermented foods have generally been characterized as safe for human consumption while many species have been recognized to confer probiotic potential [7,8].

Cheese has been reported as a suitable food carrier of probiotic bacteria. In this regard many studies have explored of the addition of probiotic strains within various cheese products targeting to study their impact on physicochemical, microbiological and sensory characteristics [9,10,11]. Feta is white brined semi-soft traditional Greek cheese of protected destination of origin (PDO) which was traditionally produced by mixtures of sheep’s and goat’s raw milk [12]. Feta cheese is one of the most important exporting products of Greece while its annual consumption within the country is estimated at 12 kg per capita [13]. It is industrially produced by pasteurized sheep’s and goat’s milk (up to 30%) in well-equipped cheese dairies, using rennin enzyme and commercial lactic acid cultures as starters. The most well-established starter culture for Feta cheese production is the commercial yogurt culture (*Lactobacillus bulgaricus* and *Streptococcus thermophilus*) while many recent studies have focused on the application of various probiotic strains as starter or adjunct cultures for Feta cheese production [12,14,15].

*L. paracasei* K5 strain, previously isolated from dairy products, was used in the present study as starter culture for Feta-type cheese production [16]. This novel strain displayed significant probiotic properties, including efficient anti-proliferative activity and adherence capacity to Caco-2 colon cancer cells [16,17]. The aim of the current study was to evaluate the use of this potential probiotic strain incorporated as a freeze-dried starter culture for functional Feta-type cheese production. The starter culture was added during industrial Feta cheese production, either as a free culture or as an immobilized synbiotic biocatalyst. The immobilized biocatalyst parted from delignified wheat bran (DWB) which was used as an immobilization carrier of *L. paracasei* K5 targeting enhanced survival of the strain during freeze-drying [18] and during cheese processing and storage conditions [3]. In addition, the produced starter cultures were incorporated during cheese production prior to and after the addition of rennin enzyme to study the optimum cheese quality characteristics.

## 2. Materials and Methods

### 2.1. Starter Cultures for Cheese Production

The recently isolated *Lactobacillus paracasei* K5, a novel potentially probiotic strain [16], was evaluated as a lactic acid bacterial starter for Feta-type cheese production. The strain was revived from Democritus University stock culture and stored at −80 °C into 10 mL of MRS broth (LabM, Heywood, United Kingdom). Subsequently, a subculture was freshly prepared in MRS broth and incubated at 37 °C for 24 h, followed by centrifugation at 12.000 g/15 min at 20 °C. 

An immobilized synbiotic biocatalyst was also prepared as a starter culture for Feta-type cheese production targeting enhanced survival rates of probiotic cells [19]. *L. paracasei* K5 was immobilized on delignified [20] wheat bran (DWB; a cereal processing prebiotic by-product) by mixing 2 g of the cell culture produced as described above with 10g of DWB in 1L of MRS broth and incubated at 37 °C for 24–48 h [12].

Both free and immobilized *L. paracasei* K5 starter cultures were subjected to freeze-drying to be assimilated to the starter cultures used by the industry [21]. Specifically, free and immobilized bacterial cells were frozen to −44 °C (cooling rate 5 °C min^−1^) and freeze-dried for 48–72 h at 5 × 10^−3^ bar and −45 °C in the Freezone 4.5 freeze-drying system (Labconco, Kansas City, Missouri, USA). No cryoprotectants were used during freeze-drying of the starter cultures [18].

### 2.2. Pilot-Scale Feta-Type Cheese Production 

**Figure 1:**
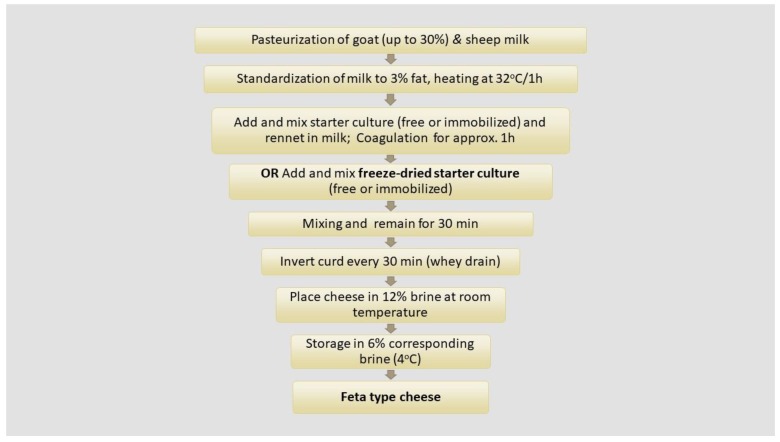
Diagram of Feta-type cheese production.

Feta-type cheeses were prepared by pasteurized and standardized sheep and goats (up to 30%) milk priory checked for the presence of antibiotics with pH determined at 6.7 ± 0.2. Pasteurization of milk was performed in a double walled stainless-steel vat at 63 °C for 30 min, after which the milk was cooled at 37 °C and standardized. All cheeses were manufactured by the addition of rennin enzyme at a proportion 0.01% *w*/*v* (Figure 1). In addition to rennin enzyme, the novel freeze-dried potential probiotic starter culture (free/immobilized) was also incorporated for cheese production. Initially the freeze-dried starter culture was added into milk prior to rennin enzyme, mixed well and allowed to rest for 30 min. For comparison reasons, the freeze-dried starter culture was also added after rennin enzyme achieved curdling for approx. 30 min. In all cases the starter cultures were suspended into 20 mL of sterile skimmed goat’s milk (0.5% fat) prior to use. The inoculum amount of the starter cultures added to milk was estimated in population levels of approx. 8 log cfu/mL. 

Pilot scale cheese production is illustrated in Figure 1. In brief, each prepared mixture was left for curd formation. Subsequently, the curd was cut and transferred into rectangular molds which were turned over at appropriate times during 24 h targeting whey draining. The following day, the curd was cut into blocks weighing approx. 500 g and each sample was placed into separate metal vessels with the addition of brine (12% *w*/*v* NaCl). Cheese ripening was performed in two stages. During the 1st ripening period cheese samples were stored for 15 days at room temperature (18–20 °C). The brine was replaced during the 2nd ripening period by adding fresh brine (6% *w*/*v* NaCl) and cheese samples remained at 4 °C for 45 days. After ripening, cheese samples (500 g) were placed into sterile plastic containers with fresh brine (6% *w*/*v* NaCl), sealed and stored at 4 °C.

Subsequently, five lots of 500 g each were prepared: Cheese 1 (C1)–commercial Feta cheese manufactured with rennin enzyme without starter culture (control), Cheese 2 (C2)–Feta-type cheese manufacture with free starter culture added prior rennin enzyme, Cheese 3 (C3)–Feta-type cheese manufacture with immobilized starter culture added prior rennin enzyme, Cheese 4 (C4)–Feta-type cheese manufacture with free starter culture added after rennin enzyme and Cheese 5 (C5)–Feta-type cheese manufacture with immobilized starter culture added after rennin enzyme. Cheese samples were collected during various time intervals of cheese ripening and storage and were evaluated regarding microbiological, physicochemical and sensory characteristics.

### 2.3. Physicochemical Analysis

The pH value of produced cheeses was determined by immersion of the electrode of a digital pH-meter (HI99161, Hanna Inc., United Kingdom) directly into the samples. Titratable acidity (%) of each cheese sample was determined by titration of 10 g of cheese suspended in 20 mL deionized water with 0.1 N NaOH using phenolphthalein as indicator [22]. Titratable acidity was expressed as lactic acid content.

For lactose determination, cheese samples (20 g) were macerated with warm (40 °C) deionized water to a total volume of 210 mL and filtrated. The filtrate was used for the determination of lactose content by high performance liquid chromatography (HPLC). The samples were filtered with disposable cellulose acetate filters (0.20 nm). The filtrates (60 μL) were injected directly into the column of a Shimadzu HPLC system which parted of a Nucleogel Ion 300 OA column, a LC-9A pump, a CTO-10A oven at 40 °C and a RID-6A refractive index detector. The mobile phase was 0.008 N H_2_SO_4_ (0.5 mL/min), while 1-propanol was used as an internal standard. Sugar concentrations were calculated using standard curves [12].

### 2.4. Scanning Electron Microscopy—SEM

Scanning electron microscopy (SEM) is a technique used for the visualization and characterization of surfaces and it was selected as imaging method because it offers a high spatial resolution and a large field of view [23]. Likewise, small particles of delignified wheat bran with immobilized *L. paracasei K5* were freeze-dried as described previously (§ 2.1) and coated with Gold (thickness 15.0 nm, density 19.32 g/cm^3^) in a Balzers SCD 004 Sputter coater (Bal-Tec, Schalksmühle, Germany) for 2–3 min. The samples were examined operating at an accelerating voltage of 20kV using a scanning electron microscope (JSM-6300, JEOL, Tokyo, Japan).

### 2.5. Microbiological Analyses

Microbiological analysis was carried out during cheese ripening and storage at various time intervals (1, 5, 10, 15, 30, 45, 55, 60, 75, 90 days). To estimate the number of viable cells on each test day, ten-gram portions of Feta-type cheese (10 g) were weighed aseptically, blended with 90 mL of sterile trisodium citrate (2% *w*/*v*) solution and homogenized in a stomacher (Bagmixer 400, Model VW, Interscience). The solution was then subjected to serial dilutions of 9 mL of Ringer solution 1/4 strength that was previously sterilized. Enumeration of viable cell counts was performed in triplicate by pour plating 0.1 mL or 1 mL of appropriate dilutions on the selective media for each species according to instructions given by the manufacturer. Specifically, total aerobic mesophilic bacteria count (TBC) were enumerated on Plate Count Agar (LabM, Heywood, United Kingdom) incubated at 30 °C for 48–72 h, lactococci (lactic streptococci) were enumerated on M17 agar (LabM, Heywood, United Kingdom) incubated at 30 °C for 48–72 h, lactobacilli (mesophilic lactic acid bacteria) were enumerated on de Man, Rogosa and Sharpe Agar–MRS agar (LabM, Heywood, United Kingdom) incubated at 37 °C for 48 h, staphylococci were enumerated on Baird Parker agar (LabM, Heywood, United Kingdom) with egg yolk tellurite medium incubated at 37 °C for 48 h, yeasts and fungi were enumerated on Potato Dextrose Agar (LabM, Lancashire, United Kingdom) incubated at 30 °C for 48–72 h, bacteria from the family *Enterobacteriaceae* were enumerated on Violet Red Bile Glucose Agar (LabM, Heywood, United Kingdom) incubated at 37 °C for 24 h and *Salmonella* spp. were enumerated on Brilliant Green (Fluka, Buchs, Switzerland) incubated at 37 °C for 24 h [12]. All cell counts were expressed as log of mean colony-forming units per gram of cheese.

### 2.6. Molecular Detection of the Starter Culture in Cheese

The survival of the new *L. paracasei* K5 strain with probiotic potential was evaluated by the end of the cheese’s storage period (4 °C) through molecular detection. Specifically, following microbial enumeration of lactobacilli in MRS agar as described before (§2.6), the petri dishes corresponding to the concentration of ≥ 6 log cfu/g were submitted for molecular analysis. In brief, each petri dish was washed with 1 mL sterilized 1/4 strength Ringer’s solution and cell suspensions were subjected to molecular analysis based on multiplex polymerase chain reaction (PCR) for the detection of *L. paracasei* K5 as described before [16]. 

### 2.7. Aromatic Volatiles Detection by SPME GC/MS 

Feta-type cheeses produced with the novel *L. paracasei* K5 as starter culture (C2, C3, C4, C5) were studied regarding their volatile profile using solid phase microextraction gas chromatography–mass spectrometry analysis (SPME GC/MS) and compared with volatile characteristics of control cheese (C1). Representative 7 g portions of cheese samples were collected from the 90th storage day (4 °C) and placed into a 20 mL headspace vial which was sealed with an aluminum crimp. The sealed glass container was initially thermostated at 60 °C for 5 min and then a syringe needle was introduced for 45 min (60 °C) through the crimp. The absorbed volatile analytes were then analyzed by solid-phase microextraction (SPME) GC-MS analysis as described previously [12]. The identification of absorbed volatiles was carried out by comparing the retention time and mass spectra of detected volatiles with those in NIST107, NIST21 and SZTERP libraries, and by determining Kovats’ retention indexes (KI) and then compared with KI reported in the literature [12,19,24]. 

### 2.8. Statistical Analysis

Each experiment was replicated twice (two independent batches at pilot scale) with three samples analyzed each time. Differences of the means for the various parameters (microbiological and chemical) were tested by using the Analysis of Variance One-way (ANOVA) procedure with Tukey’s HSD *post hoc* application for multiple comparisons at a 95% confidence level to discriminate among the means of the various samples. Statistical analysis was performed with IBM^®^ SPSS^®^ v20. The microbial counts obtained from the analysis of C1 to C5 group of cheese samples were logarithmically (LOG10) transformed and presented along with their respective standard deviations. Accordingly, the means and standard deviations from three replicates of the GC-MS analyses are also presented. 

## 3. Results and Discussion 

### 3.1. Physicochemical Characteristics of Feta-Type Cheese

**Figure 2:**
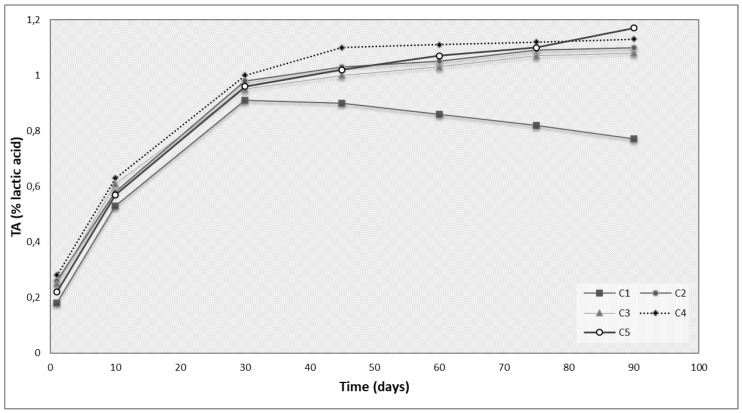
Titratable acidity (% lactic acid) changes in Feta-type cheese during ripening for 60 days and storage for 30 days at 4 °C. C1–commercial Feta cheese manufactured with rennin enzyme, C2–Feta-type cheese manufacture with free starter culture added prior rennin enzyme, C3–Feta-type cheese manufacture with immobilized starter culture added prior rennin enzyme, C4–Feta-type cheese manufacture with free starter culture added after rennin enzyme, C5–Feta-type cheese manufacture with immobilized starter culture added after rennin enzyme.

**Figure 3:**
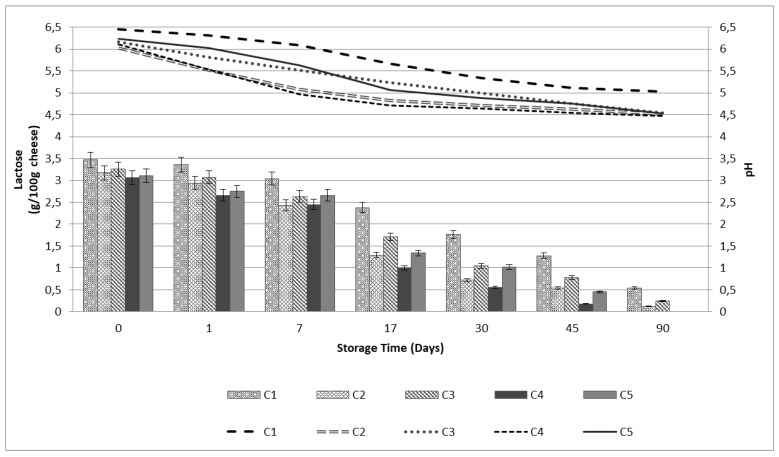
pH and lactose content (in g per 100 g cheese, on wet weight basis) of Feta-type cheeses during 60 days of ripening and 30 days of storage. C1–commercial Feta cheese manufactured with rennin enzyme, C2–Feta-type cheese manufacture with free starter culture added prior rennin enzyme, C3–Feta-type cheese manufacture with immobilized starter culture added prior rennin enzyme, C4–Feta-type cheese manufacture with free starter culture added after rennin enzyme, C5–Feta-type cheese manufacture with immobilized starter culture added after rennin enzyme.

The changes of the mean values of pH, lactose and total acidity (%) during ripening and storage for 90 days are presented in Figure 2 and Figure 3. In general, all parameters ranged in levels usually observed in Feta-type cheese products [12,14,15]. There was an increased titratable acidity observed (Figure 2) in parallel with the decreasing of pH (Fig. 3) in all trials during the 1st ripening period. Similar results were also observed during the 2nd ripening period of cheese samples. On the other hand, during cold storage (60th–90th day) of cheese samples (4 °C), titratable acidity differentiated among trials. Specifically, titratable acidity was found to be increased in all samples prepared by the use of *L. paracasei* K5 as starter culture (C2, C3, C4, C5) ranging between 1.03~1.17 (% lactic acid) while in the case of control cheese (C1) titratable acidity ranged between 0.86~0.77 (Figure 2). These findings indicate that the use of lactic acid bacteria in cheese production can increase acidity of cheese, even during cold storage (4 °C) [10,14].

Figure 3 demonstrates the changes in the pH and lactose content during ripening and storage time. A strong interaction was observed between the starter culture and ripening time affecting lactose concentration and pH. The part of lactose which remained in cheese after curd draining decreased in all cases during ripening and storage (Figure 3). Lactose was catabolized by the starter culture as well as non-starter lactic acid bacteria and other microorganisms mainly during the 1st ripening period [15]. As a result, in the case of Feta-type cheese produced with immobilized *L. paracasei* K5 (C4, C5) we can observe that no lactose content was detected by the end of the storage period (90th day), while in the case of Feta-type cheese produced with *L. paracasei* K5 free cells (C2, C3) only traces of lactose were detected. The use of the novel starter culture significantly affected lactose content compared to control cheese samples (C1) during cheese ripening and storage. Subsequently, all cheese samples manufactured with *L. paracasei* K5 starter culture (C2, C3, C4, C5) were characterized by a significantly (*p* < 0.05) lower pH and lactose content, compared to control cheese samples (C1). This result is in accordance with previous studies that reported significant lactose reduction during the 1st period of Feta-type cheese ripening [12,15]. 

### 3.2. Microbiological Profile of Cheese Products

The microbiological characteristics of produced cheeses was carried out by plate counting during ripening and storage for 90 days in total and the results are presented in Table 1. 

During cheese manufacture it is of prime importance that a rapid conversion of lactose is achieved by starter microorganisms in parallel with the concomitant lowering of pH in order to inhibit the growth of undesired microorganisms during cheese manufacture [21]. According to our results, there was a statistically significant difference between the means of total bacteria counts (TBC) counts among the groups C1–C5 (ANOVA F-test = 3.22. *p* = 0.014). Differences were also observed for lactobacilli (ANOVA F-test = 172.16. *p* = 0.00). Lactococci (ANOVA F-test = 16.42, *p* = 0.00), enterobacteria (ANOVA F-test = 31.49, *p* = 0.00), yeasts & fungi (ANOVA F-test = 11.27, *p* = 0.00) and staphylococci (ANOVA F-test = 7.41, *p* = 0.00). More specifically, a small population of enterobacteria and staphylococci were detected in all cheese samples during the 1st ripening period. The detection of enterobacteria and staphylococci are usually expected in white brined cheese production as these microbes are used as an index of animal health (e.g., mastitis) or as an index of the hygiene practices during the milking and later handling of milk [25]. These microorganisms can be sensitive to low pH values and thus the use of a starter culture is mandatory, especially in the case of raw unpasteurized milk with an increased load of microorganisms. In our study it is reported that by the addition of *L. paracasei* K5 as starter culture in all cases the initial loads of enterobacteria and staphylococci were reduced by the end of the 2nd ripening period. More importantly, in the case of cheese produced by the immobilized biocatalyst (C4, C5), no enterobacteria were detected during cheese storage at 4 °C. In general, enterobacteria are considered as the most abundant group of microorganisms detected in milk after non-starter mesophilic lactobacilli [26] and it is crucial that they are reduced by the 1st ripening period. In the present study, there a significant decrease was detected in cheese samples produced by *L. paracasei* K5 starter while a complete absence of enterobacteria counts was observed after the 60th day of production in all cases (C2, C3, C4, C5). On the other hand, enterobacteria in control cheese (C1) were still detected up to the 90th day of storage. A possible explanation for this observation could be the high population of the starter culture which through the production of bacteriocins or antagonism in nutrients can lead to the reduction of spoilage or other possible pathogenic microorganisms in milk products [26,27,28]. Thus, it is highlighted that an efficient starter culture is mandatory during cheese ripening targeting a fast pH decrease, as was provided by *L. paracasei* K5 starter culture. Such an observation has also been reported in other studies using probiotic strains for cheese production [10,26,29]. The increased population of the starter culture can also be assumed by the high lactobacilli counts detected in cheese samples C2, C3, C4 and C5 during ripening and storage compared with cheese samples produced with no starter culture (C1). On the other hand, lactococci which are considered indigenous milk microbiota did not significantly differ (*p* > 0.05) among cheese samples. Finally, no *Staphylococcus aureus* or *Salmonella* spp. was detected in any of our cheese samples during 90 days of ripening and storage.

Another significant group of microorganisms regarding cheese microbiological profile is yeast and fungi. In general, yeast and fungi are considered to be predominant microbiota of milk products; in most cases they can be detected during cheese ripening while in some cases they can be even used as starter cultures for cheese manufacture [30,31]. When yeasts and fungi are not used as starters for cheese production, they are considered unwanted since they metabolize organic acids, causing an increase in pH and as a result providing a friendly environment for the growth of other spoilage and pathogenic bacteria [31]. A relatively high number of yeast and fungi was detected in all samples, most likely as a result of contamination by industry environment; originating from cheesemaking equipment or cheese brine [32]. In the present study, yeast and fungi count were reduced in all samples after the 2nd ripening period and were detected as less abundant in Feta-type cheeses produced with *L. paracasei* K5 starter culture. These findings are in accordance with other studies reporting that lactobacilli can provide an antifungal activity in cheese [9,33].

### 3.3. Survival of L. Paracasei K5 in Feta-Type Cheese during Storage

**Figure 4:**
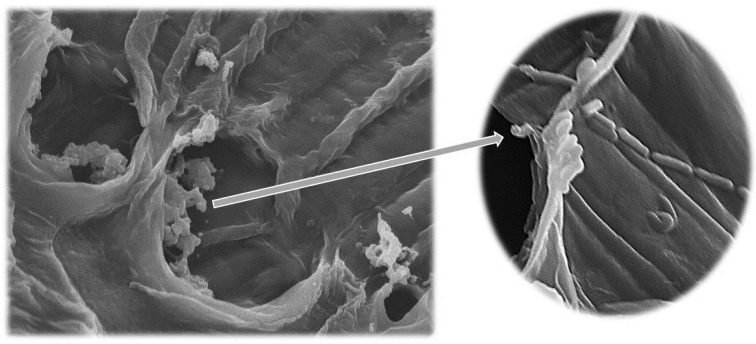
Electron micrographs of the freeze-dried immobilized on DWB *L. paracasei* K5 (left: × 30 μm and right × 10 μm).

In probiotic cheese products, survival of lactic acid bacteria may be reduced due to the high salt concentration, antagonism of other microorganisms and pH [34]. While targeting to provide high survival rates of the potential probiotic starter, the culture was not only introduced as a free freeze-dried *L. paracasei* K5 starter but also as an immobilized synbiotic biocatalyst [12,19]. The suitability of wheat bran as immobilization carrier has been demonstrated by previous studies [19,35] in addition to its ability to deliver probiotic lactic acid bacteria to the human gut [36,37]. Subsequently, the potential probiotic *L. paracasei* K5 was successfully immobilized in DWB as illustrated in Figure 4. 

Nowadays, starter cultures are largely produced by commercial companies and they provide an extensive range of frozen and freeze-dried concentrated cultures [21]. In the present study, both free and immobilized bacterial starter cultures had been submitted to free-drying targeting assimilation with industrial starters. In addition, bacterial cultures were introduced during cheese manufacture prior and after rennin enzyme in order to investigate the starter cultures’ survival capacity and technological characteristics. 

**Figure 5:**
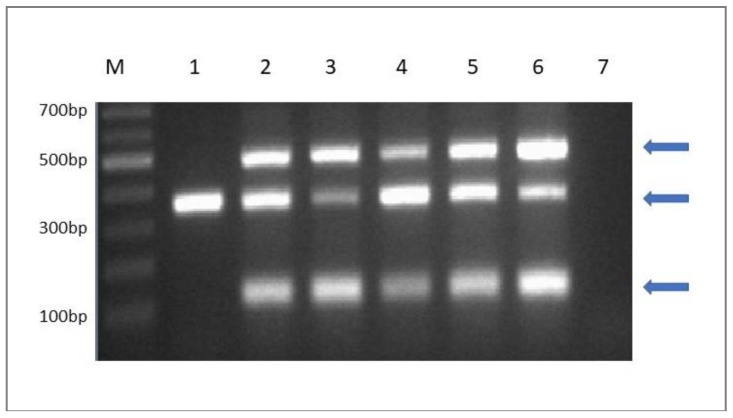
Molecular identification of *L. paracasei* K5 at levels ≥ 6 log cfu/g in Feta-type cheese after 90th day of production by strain-specific multiplex PCR assay (M: DNA ladder, Lane 1: C1 control cheese, Lane 2: C2 cheese, Lane 3: C3 cheese, Lane 4: C4 cheese Lane 5: C5 cheese, Lane 6: pure culture of Lb. paracasei K5 and Lane 7: negative control).

The results from microbiological enumeration and multiplex PCR analysis showed that *L. paracasei* K5 was detected in all cheese products (samples C2, C3, C4, C5) manufactured by the use of the starter culture, at levels of at least 6 log cfu/g (Figure 5). As expected, *L. paracasei* K5 was not detected in control samples (C1). These values comply with the main requirement for probiotic foods regarding a minimum population of 6 log cfu/g live probiotic cells at the moment of consumption [38]. Recently *L. paracasei* K5 was reported to display efficient adherence capacity to Caco-2 colon cancer cells, similarly to the reference strains *Lactobacillus casei* ATCC 393 and *Lactobacillus rhamnosus* GG [17]. As a result, it is demonstrated that *L. paracasei* K5 can be successfully used as starter culture for Feta-type cheese production, retain its high survival rates during storage and possibly provide beneficial effects to the consumer. In addition, the use of the immobilized biocatalyst (C3, C5) in cheese production can provide the consumer with a novel potential probiotic cheese assembled with prebiotic constituents [36,39].

### 3.4. Impact of the Starter Culture on Aromatic Characteristics of Cheese

During cheese manufacture, various biochemical reactions take place simultaneously especially during cheese ripening that have a crucial impact on cheese aromatic profile. It has been established that cheese aromatic characteristics do not depend on particular key components, but on a weighted concentration ratio of total by-products formation [40]. These aroma related compounds have the most important impact on the final quality of cheese and thus on its acceptance during consumption. The majority of volatiles that define a characteristic aroma to maturated Feta-type cheeses after 30 days of storage at 4 °C are presented in Table 2, classified by chemical families (esters, free fatty acids, alcohols, carbonyl compounds and lactones).

One of the most important volatile compounds responsible for cheese aromatic characteristics are esters. In general, esters are most desirable among volatiles as they have low threshold values, conferring an important impact even at low concentrations while they usually provide floral or fruity notes, which smoothens other undesirable odors [40]. In the present study the esters detected to be responsible for a fruity or floral odor in cheese samples are ethyl butanoate, ethyl hexanoate, methyl octanoate, ethyl decanoate and 2-phenylethyl acetate. Specifically, ethyl hexanoate was detected to be more abundant in cheese samples produced by the immobilized biocatalyst (C4>C5) and is known to provide wine-like, brandy or valeriana-like notes to cheese products [41]. Methyl octanoate was found to be almost equivalent in all cheese samples produced by *L. paracasei* K5 starter culture (C2, C3, C4, C5) while it was not detected in control cheese samples (C1). It has been reported that methyl octanote can provide flower-like, caramel-like or valeriana-like notes, resulting in a more smooth-like cheese flavor [41]. Likewise, ethyl octanoate is known to provide a pleasant, fruity, floral, fresh or even sweet essence in cheese products and has been detected to be most abundant in cheese samples produced with the starter culture (C2, C3, C4, C5). A similar ester providing fruity, grape or even brandy-like notes to cheese products has been reported to be ethyl decanoate, which was also detected to be most abundant in cheese samples produced by the starter culture (C2, C3, C4, C5) [41]. An overall comparison between cheese samples and ester by-products formation shows an enhanced content in samples produced by the immobilized biocatalyst (C4, C5) followed by samples produced with free starter culture (C2, C3) and then samples produced with no starter culture (C1) which suppress of lower concentration. Therefore, it can be concluded that *L. paracasei* K5 starter culture can provide cheese products enhanced by sweet floral or fruity notes which can be characterized by a smoother and more unique flavor.

Another category of major significance regarding the aromatic profile of cheese is free fatty acids. Free fatty acids contribute to cheese flavor either by their aromatic notes or by a rancidity defect when they occur in very large amounts [42]. Short and medium length free fatty acids (C4:0-C12:0) are characterized by low threshold values and thus can provide a characteristic flavor to the final product [42]. In the present study, the most abundant detected free fatty acids were butanoic, hexanoic, octanoic and decanoic acid, which are generally known to provide a characteristic milk flavor to cheese products [43]. In the present study, short and medium length free fatty acids were detected to be most abundant in cheese samples produced with *L. paracasei* K5 starter culture (C2, C3, C4, C5). These fatty acids are known to be mainly produced through accumulation of lipase enzyme on milk fat or from the breakdown of amino acids [44,45]. Since lipase enzyme was added in all cheese samples in equivalent amounts, we can assume that the starter culture enhanced the production of acids in the samples. 

The produced hexanoic acid which was detected to be significantly higher in cheese samples produced by the starter culture (C2, C3, C4, C5) compared with control cheese (C1) is reported to provide sweaty, sour, sharp pungent, cheesy, or fatty notes to cheese products. On the other hand, decanoic acid which was detected in equivalent amount in all cheese products, is usually responsible for an unpleasant rancid, fatty or sometimes a weak terpenic citrus-like character [41]. The unpleasant characteristics those kinds of acids can be trespassed when combined with other volatiles [46]. 

Ethanol, which is a compound identified in many cheese products was also detected to be most abundant in all cheese samples of the present study. Ethanol is considered to provide a mild flavor note and may also have derived by the metabolic activity of the starter culture or yeasts present during cheese ripening (Table 1) [10,12]. Finally, lactones are known to have a similar impact to esters as they also provide very low threshold values and are characterized by fruity, creamy and buttery attributes [47]. In all cheese samples δ-decalactone and δ-dodecalactone were detected. The most abundant among lactones was reported to be δ-decalactone, which has been reported to provide creamy and buttery notes to cheese products [47]. In general, Feta-type cheeses produced by the immobilized biocatalysts (C4, C5) were reported to be most abundant regarding lactones formation while γ- dodecalactone was only detected in these cheese samples. Finally, the results of volatiles analysis revealed that many aromatic compounds were significantly enhanced by the novel starter culture (free or immobilized), highlighting its potential industrial use as a ready-to-use starter.

## 4. Conclusions

*L. paracasei* K5 was successfully submitted (free or immobilized) as a starter culture for Feta-type cheese production. In addition, its possible probiotic effects can provide a novel functional cheese product to the consumer since high survival levels (≥6.0 log cfu/g) were verified by combining microbiological enumeration and strain-specific multiplex PCR analysis even after the 90th day of production. Volatile evaluation revealed cheese products of enhanced aromatic characteristics revealed by the use of *L. paracasei* K5 starter culture. Regarding the use of the immobilized biocatalyst, it is of major importance that the immobilization technique is of low cost, no cryoprotectants were used during freeze-drying and cheese products were found to be enhanced in aromatic characteristics in addition to a decreased number of possible spoilage or pathogenic microorganisms. These findings may contribute to the development of standard starter cultures for Feta-type cheese production with improved nutritional value. Finally, the traditional production of Feta cheese can be revitalized considering consumers’ concerns regarding safety and health attributes by the use of novel functional starter cultures which can produce cheese with improved quality characteristics and in parallel preserve its unique identity.

## Figures and Tables

**Table 1:** Microbial populations (log cfu/g) of total aerobic counts, enterobacteria, yeasts & fungi, lactococci and lactobacilli in Feta-type cheeses during 60 days of ripening and 30 days of storage at 4 °C.

	Time (days)	C1	C2	C3	C4	C5
TBC (log cfu/g)	1	7.1 ± 0.1 ^a^	7.7 ± 0.2 ^b^	8 ± 0.1 ^bc^	8.57 ± 0.06 ^d^	8.27 ± 0.25 ^cd^
	5	8.4 ± 0.1 ^a^	7.67 ± 0.06 ^b^	8.3 ± 0.1 ^a^	8.2 ± 0.2 ^a^	8.2 ± 0.2 ^a^
	10	8.33 ± 0.06 ^a^	8.23 ± 0.06 ^a^	7.9 ± 0.1 ^b^	8.03 ± 0.06 ^b^	8.53 ± 0.06 ^c^
	15	8.83 ± 0.06 ^a^	7.8 ± 0.1 ^b^	7.47 ± 0.06 ^c^	8.1 ± 0.1 ^d^	8.23 ± 0.06 ^d^
	30	8.37 ± 0.21 ^a^	8.13 ± 0.15 ^ab^	7.87 ± 0.06 ^bc^	7.67 ± 0.06 ^c^	7.73 ± 0.06 ^c^
	45	8.1 ± 0.1 ^a^	7.9 ± 0.1 ^ab^	7.7 ± 0.1 ^bc^	7.6 ± 0 ^bc^	7.5 ± 0.2 ^c^
	55	8 ± 0.1 ^a^	7.5 ± 0.2 ^b^	7.37 ± 0.06 ^bd^	6.33 ± 0.06 ^c^	7.17 ± 0.06 ^d^
	60	7.4 ± 0.2 ^a^	6.87 ± 0.06 ^b^	6.77 ± 0.06 ^b^	5.6 ± 0.1 ^c^	6.67 ± 0.06 ^b^
	75	7.03 ± 0.06 ^a^	6.5 ± 0.17 ^b^	6.67 ± 0.12 ^b^	5.23 ± 0.15 ^c^	6.1 ± 0.1 ^d^
	90	6.67 ± 0.06 ^a^	6.7 ± 0.1 ^a^	6.13 ± 0.06 ^b^	4.63 ± 0.31 ^c^	5.8 ± 0.1 ^b^
Count of *Lactobacillus* genus bacteria (log cfu/g)	1	5.23 ± 0.15 ^a^	8.1 ± 0.1 ^b^	7.63 ± 0.06 ^c^	8.5 ± 0.1 ^d^	8.2 ± 0.1 ^b^
	5	5.9 ± 0.1 ^a^	8.3 ± 0.1 ^b^	8.13 ± 0.15 ^b^	8.7 ± 0.1 ^c^	8.63 ± 0.06 ^c^
	10	6.1 ± 0.1 ^a^	8.6 ± 0.1 ^bc^	8.3 ± 0.1 ^b^	8.8 ± 0.2 ^c^	8.5 ± 0.1 ^bc^
	15	6.2 ± 0.1 ^a^	8.5 ± 0.1 ^bc^	8.4 ± 0.1 ^b^	8.6 ± 0.1 ^bc^	8.7 ± 0.1 ^c^
	30	5.7 ± 0.3 ^a^	8.6 ± 0.2 ^b^	7.9 ± 0.1 ^c^	8.6 ± 0.1 ^b^	8.5 ± 0.1 ^b^
	45	5.4 ± 0.1 ^a^	8.7 ± 0.1 ^b^	8.03 ± 0.06 ^c^	8.37 ± 0.06 ^d^	8.3 ± 0.1 ^d^
	55	5.47 ± 0.06 ^a^	8.17 ± 0.06 ^bc^	7.7 ± 0.1 ^d^	8.33 ± 0.06 ^c^	8.03 ± 0.06 ^b^
	60	5.2 ± 0.1 ^a^	7.7 ± 0.1 ^b^	7.5 ± 0.1 ^b^	8.5 ± 0.1 ^c^	7.7 ± 0.1 ^b^
	75	4.67 ± 0.15 ^a^	7.4 ± 0.1 ^b^	7.2 ± 0.2 ^b^	8.03 ± 0.06 ^c^	7.5 ± 0.1 ^b^
	90	4.2 ± 0.2 ^a^	7.1 ± 0.1 ^b^	6.7 ± 0.1 ^c^	7.7 ± 0.1 ^d^	7.1 ± 0.2 ^b^
Count of *Lactococcus* genus bacteria (log cfu/g)	1	7.7 ± 0.2 ^a^	7.3 ± 0.2 ^b^	7.6 ± 0.1 ^ab^	6.7 ± 0.1 ^c^	7.7 ± 0.1 ^a^
	5	8.2 ± 0.1 ^a^	7.7 ± 0.1 ^b^	7.4 ± 0.2 ^b^	6.4 ± 0.1 ^c^	7.4 ± 0.1 ^b^
	10	7.9 ± 0.1 ^a^	7.5 ± 0.1 ^b^	6.73 ± 0.06 ^c^	6.2 ± 0.1 ^d^	7.6 ± 0.1 ^b^
	15	7.6 ± 0.2 ^a^	7.8 ± 0.1 ^a^	6.3 ± 0.1 ^b^	5.9 ± 0.1 ^c^	7.2 ± 0.1 ^d^
	30	7.5 ± 0.1 ^a^	6.6 ± 0.1 ^b^	6.4 ± 0.1 ^b^	5.9 ± 0.1 ^c^	6.6 ± 0.1 ^b^
	45	7.7 ± 0.1 ^a^	6.8 ± 0.1 ^b^	6.4 ± 0.1 ^c^	4.6 ± 0.2 ^d^	5.27 ± 0.06 ^e^
	55	7.4 ± 0.1 ^a^	6.1 ± 0.1 ^b^	4.97 ± 0.15 ^c^	4.1 ± 0.1 ^c^	4.73 ± 0.06 ^d^
	60	6.93 ± 0.06 ^a^	5.8 ± 0.2 ^b^	4.7 ± 0.1 ^c^	3.7 ± 0.1 ^d^	4.1 ± 0.1 ^e^
	75	6.5 ± 0.1 ^a^	5.5 ± 0.1 ^b^	4.1 ± 0.1 ^c^	3.2 ± 0.2 ^d^	3.7 ± 0.1 ^e^
	90	6.13 ± 0.06 ^a^	5.1 ± 0.1 ^b^	4 ± 0.2 ^c^	3 ± 0.1 ^d^	3.33 ± 0.06 ^e^
Count of *Enterobacteriaceae* family bacteria (log cfu/g)	1	2.9 ± 0.1 ^a^	2.4 ± 0.2 ^b^	2.2 ± 0.2 ^bc^	1.9 ± 0.1 ^c^	2.4 ± 0.1 ^b^
	5	4.8 ± 0.1 ^a^	2.2 ± 0.1 ^b^	2.27 ± 0.15 ^b^	2.3 ± 0.1 ^bc^	2.6 ± 0.1 ^c^
	10	5.3 ± 0.2 ^a^	3.6 ± 0.1 ^b^	2.7 ± 0.1 ^c^	1.6 ± 0.2 ^d^	2.9 ± 0.1 ^c^
	15	4.4 ± 0.2 ^a^	3.1 ± 0.1 ^b^	2 ± 0.2 ^c^	1.8 ± 0.2 ^c^	2.2 ± 0.4 ^c^
	30	4.1 ± 0.1 ^a^	2.7 ± 0.1 ^b^	2.27 ± 0.25 ^c^	1.03 ± 0.06 ^d^	1.6 ± 0.1 ^e^
	45	3.6 ± 0.1 ^a^	2.1 ± 0.1 ^b^	1.7 ± 0.1 ^c^	nd	1.07 ± 0.06 ^d^
	55	3.3 ± 0.1 ^a^	1.6 ± 0.1 ^b^	1.6 ± 0.1 ^b^	nd	nd
	60	2.8 ± 0.2 ^a^	1.3 ± 0.1 ^b^	1.3 ± 0.2 ^b^	nd	nd
	75	2.6 ± 0.1	nd		nd	nd
	90	2.4 ± 0.1	nd	nd	nd	nd
Count of Yeasts & Fungi (log cfu/g)	1	6.4 ± 0.1 ^a^	5.8 ± 0.2 ^b^	6.6 ± 0.1 ^a^	6.7 ± 0.1 ^a^	6.6 ± 0.1 ^a^
	5	5.7 ± 0.1 ^a^	5.1 ± 0.1 ^b^	6.2 ± 0.1 ^c^	6.2 ± 0.1 ^c^	7.2 ± 0.2 ^d^
	10	6.1 ± 0.1 ^a^	5.3 ± 0.2 ^b^	5.7 ± 0.1 ^c^	6 ± 0.1 ^ac^	7 ± 0.1 ^d^
	15	5.8 ± 0.1 ^a^	5.2 ± 0.1 ^b^	6.1 ± 0.1 ^c^	6.03 ± 0.06 ^ac^	6.8 ± 0.1 ^d^
	30	5.6 ± 0.1 ^a^	4.8 ± 0.1 ^b^	5.4 ± 0.1 ^a^	5.7 ± 0.1 ^a^	6.2 ± 0.2 ^c^
	45	5.9 ± 0.2 ^a^	4.1 ± 0.1 ^b^	5.2 ± 0.1 ^c^	5.2 ± 0.1 ^c^	5.7 ± 0.1 ^a^
	55	6.2 ± 0.1 ^a^	4.1 ± 0.1 ^b^	4.9 ± 0.1 ^c^	5.03 ± 0.06 ^c^	5.1 ± 0.1 ^c^
	60	5.9 ± 0.1 ^a^	3.8 ± 0.2 ^b^	4.4 ± 0.2 ^c^	4.3 ± 0.1 ^c^	4.9 ± 0.1 ^d^
	75	5.4 ± 0.2 ^a^	3.4 ± 0.2 ^b^	4.2 ± 0.2 ^c^	3.6 ± 0.1 ^b^	4.6 ± 0.1 ^c^
	90	4.8 ± 0.1 ^a^	3.1 ± 0.1 ^b^	3.7 ± 0.1 ^b^	3.2 ± 0.1 ^c^	4.4 ± 0.1 ^d^
Count of *Staphylococcus* genus bacteria (log cfu/g)	1	1.1 ± 0.1 ^a^	1.4 ± 0.2 ^b^	1.0 ± 0.0 ^a^	1.1 ± 0.1 ^ab^	1.2 ± 0.1 ^ab^
	5	1.7 ± 0.1 ^ab^	1.3 ± 0.1 ^c^	1.3 ± 0.2 ^c^	1.4 ± 0.1 ^ac^	1.9 ± 0.1 ^b^
	10	1.4 ± 0.1 ^a^	1.1 ± 0.1 ^b^	1.4 ± 0.1 ^a^	1.1 ± 0.1 ^b^	1.7 ± 0.1 ^c^
	15	1.27 ± 0.25 ^ab^	1.0 ± 0.0 ^a^	1.6 ± 0.1 ^b^	1.0 ± 0.0 ^a^	2.1 ± 0.1 ^c^
	30	1.1 ± 0.1 ^a^	nd	1.2 ± 0.1 ^ac^	1.03 ± 0.06 ^a^	1.4 ± 0.1 ^c^
	45	1.0 ± 0.0 ^a^	nd	1.0 ± 0.0 ^a^	nd	1.1 ± 0.1 ^a^
	55	1.1 ± 0.1 ^a^	nd	nd	nd	1.0 ± 0.0 ^a^
	60	1.0 ± 0.0	nd	nd	nd	nd
	75	1.0 ± 0.0	nd	nd	nd	nd
	90	1.0 ± 0.0	nd	nd	nd	nd

* nd. viable cells not detected. ^*^ C1–commercial Feta cheese manufactured with rennin enzyme, C2–Feta-type cheese manufacture with free starter culture added prior rennin enzyme, C3–Feta-type cheese manufacture with immobilized starter culture added prior rennin enzyme, C4–Feta-type cheese manufacture with free starter culture added after rennin enzyme, C5–Feta-type cheese manufacture with immobilized starter culture added after rennin enzyme. ^**a**–e^ Similar superscript letters in each row indicates no difference between the mean microbial counts for samples C1 to C5 (ANOVA with Tukey’s HSD *post hoc* application *at 95% confidence level*).

**Table 2:** Major aroma-related compounds (μg/kg of cheese) detected in Feta-type cheeses during refrigerated storage (30th day of storage at 4 °C) by solid-phase microextraction (SPME) GC-MS technique.

Compound	Identification Methods ^α^	KI ^β^	C1 ^*^	C2 ^*^	C3 ^*^	C4 ^*^	C5 ^*^
*Esters*							
Ethyl butanoate	MS, KI	1041	1.07 ± 0.03 ^a^	0.17 ± 0.02 ^b^	0.26 ± 0.02 ^c^	0.10 ± 0.03 ^b^	0.19 ± 0.05 ^bc^
Ethyl hexanoate	MS, KI	1251	0.00 ± 0.00 ^a^	0.14 ± 0.03 ^b^	0.11 ± 0.02 ^b^	2.60 ± 0.04 ^c^	2.16 ± 0.04 ^d^
Hexyl acetate	MS, KI	1284	0.00 ± 0.00 ^a^	0.00 ± 0.00 ^a^	0.00 ± 0.00 ^a^	0.44 ± 0.58 ^a^	0.08 ± 0.02 ^a^
Methyl octanoate	MS, KI	1380	0.00 ± 0.00 ^a^	1.14 ± 0.02 ^b^	1.36 ± 0.02 ^c^	1.26 ± 0.03 ^d^	1.04 ± 0.04 ^e^
Ethyl octanoate	MS, KI	1421	1.06 ± 0.01 ^a^	5.24 ± 0.05 ^b^	6.00 ± 0.05 ^c^	6.72 ± 0.02 ^d^	6.45 ± 0.04 ^e^
Ethyl decanoate	MS, KI	1634	8.84 ± 0.05 ^a^	16.70 ± 0.60 ^b^	12.54 ± 0.05 ^c^	26.15 ± 0.05 ^d^	20.25 ± 0.05 ^e^
2-Phenylethyl acetate	MS, KI	1830	1.35 ± 0.03 ^a^	3.03 ± 0.02 ^b^	4.15 ± 0.05 ^c^	3.07 ± 0.07 ^b^	2.23 ± 0.04 ^d^
Ethyl dodecanoate	MS, KI	1848	7.15 ± 0.06 ^a^	11.15 ± 0.09 ^b^	10.48 ± 0.03 ^c^	0.00 ± 0.00 ^d^	0.00 ± 0.00 ^d^
*Free Fatty acids*							
Butanoic acid (C4:0)	MS, KI	1642	76.16 ± 0.06 ^a^	87.14 ± 0.11 ^b^	79.08 ± 1.85 ^c^	68.74 ± 0.27 ^d^	87.46 ± 0.49 ^b^
Hexanoic acid (C6:0)	MS, KI	1851	121.33 ± 11.50 ^a^	385.73 ± 5.56 ^b^	273.63 ± 3.26 ^c^	391.97 ± 3.78 ^b^	385.83 ± 2.44 ^b^
Octanoic acid (C8:0)	MS, KI	2064	70.73 ± 5.40 ^a^	187.10 ± 6.16 ^b^	167.33 ± 3.58 ^c^	205.00 ± 6.52 ^d^	192.40 ± 3.12 ^bd^
Decanoic acid (C10:0)	MS, KI	2336	189.13 ± 3.65 ^a^	267.70 ± 2.72 ^b^	243.23 ± 1.36 ^c^	314.00 ± 4.33 ^d^	239.20 ± 8.86 ^c^
Dodecanoic acid (C12:0)	MS, KI	2485	67.57 ± 5.27 ^a^	79.54 ± 2.75 ^b^	78.93 ± 5.42 ^ab^	88.15 ± 5.51 ^b^	87.69 ± 2.01 ^b^
*Alcohols*							
ethanol	MS, KI	932	>10.000	"	"	"	"
3-Methyl-1-butanol	MS, KI	1216	0.00 ± 0.00 ^a^	4.82 ± 0.30 ^b^	4.22 ± 0.06 ^c^	6.18 ± 0.03 ^d^	5.25 ± 0.10 ^e^
1-Hexanol	MS, KI	1363	1.01 ± 0.04 ^a^	0.00 ± 0.00 ^b^	0.00 ± 0.00 ^b^	0.00 ± 0.00 ^b^	0.00 ± 0.00 ^b^
1-octen-3-ol	MS, KI	1457	0.00 ± 0.00 ^a^	0.18 ± 0.07 ^a^	0.50 ± 0.57 ^b^	0.00 ± 0.00 ^a^	0.00 ± 0.00 ^a^
1-Octanol	MS, KI	1555	0.00 ± 0.00 ^a^	1.25 ± 0.05 ^b^	1.11 ± 0.03 ^b^	3.17 ± 0.10 ^c^	3.11 ± 0.19 ^c^
2,3 butanediol	MS, KI	1569	1.04 ± 0.04 ^a^	55.37 ± 93.21 ^a^	1.67 ± 0.05 ^a^	4.45 ± 2.92 ^a^	4.07 ± 0.05 ^a^
Phenyl ethanol	MS, KI	1932	18.40 ± 0.54 ^a^	7.16 ± 0.53 ^b^	10.03 ± 0.14 ^c^	8.07 ± 0.05 ^b^	12.29 ± 0.48 ^d^
*Carbonyl compou0s*							
propanal	MS	<800	0.11 ± 0.02 ^a^	0.00 ± 0.00 ^b^	0.00 ± 0.00 ^b^	0.00 ± 0.00 ^b^	0.00 ± 0.00 ^b^
hexanal	MS, KI	1088	0.00 ± 0.00 ^a^	0.00 ± 0.00 ^a^	0.00 ± 0.00 ^a^	0.12 ± 0.06 ^b^	0.00 ± 0.00 ^a^
octanal	MS, KI	1301	0.00 ± 0.00 ^a^	0.14 ± 0.01 ^b^	0.26 ± 0.05 ^c^	0.18 ± 0.01 ^b^	0.73 ± 0.04 ^d^
3-hydroxy,2-butanone	MS, KI	1310	0.00 ± 0.00 ^a^	2.27 ± 0.06 ^b^	2.67 ± 0.04 ^c^	1.25 ± 0.04 ^d^	3.13 ± 0.09 ^e^
Nonanal	MS, KI	1395	1.75 ± 0.03 ^a^	6.15 ± 0.12 ^b^	6.77 ± 0.24 ^b^	8.56 ± 0.57 ^c^	7.57 ± 0.02 ^d^
Benzaldehyde	MS, KI	1528	2.15 ± 0.12 ^a^	0.81 ± 0.01 ^b^	1.22 ± 0.06 ^c^	0.15 ± 0.04 ^d^	2.14 ± 0.07 ^a^
*Lactones*							
δ-decalactone	MS, KI	2209	1.05 ± 0.04 ^a^	12.16 ± 0.06 ^b^	12.84 ± 0.61 ^bc^	15.46 ± 1.53 ^c^	11.91 ± 1.88 ^b^
γ- dodecalactone	MS, KI	2388	0.00 ± 0.00 ^a^	0.00 ± 0.00 ^a^	0.00 ± 0.00 ^a^	3.15 ± 0.04 ^b^	2.82 ± 0.57 ^b^
δ-dodecalactone	MS, KI	2437	3.05 ± 0.05 ^a^	10.14 ± 0.12 ^b^	8.07 ± 0.65 ^c^	10.73 ± 1.17 ^b^	10.12 ± 0.06 ^b^

**^a^** Methods of identification: **KI** = tentative identification by Kovats retention index, **MS** = tentative identification by mass spectra obtained from NIST107, NIST21, and SZTERP libraries [CLASS 5000 software of GC-17A/QP-5050A (GC-MS); Shimadzu Corp., Kyoto, Japan]. **^β^ KI** = Kovats retention index in accordance with the literature [9,10,12,39]. ^*^ C1–commercial Feta cheese manufactured with rennin enzyme, C2–Feta-type cheese manufacture with free starter culture added prior rennin enzyme, C3–Feta-type cheese manufacture with immobilized starter culture added prior rennin enzyme, C4–Feta-type cheese manufacture with free starter culture added after rennin enzyme, C5–Feta-type cheese manufacture with immobilized starter culture added after rennin enzyme. **^a–e^** Similar superscript letters in each row indicates no difference between the means of various compounds for samples C1 to C5 (ANOVA with Tukey’s HSD *post hoc* application *at 95% confidence level*).
